# Health Benefits Beyond the Scale: The Role of Diet and Nutrition During Weight Loss Programmes

**DOI:** 10.3390/nu16213585

**Published:** 2024-10-22

**Authors:** Francisca Contreras, Werd Al-Najim, Carel W. le Roux

**Affiliations:** UCD Conway Institute of Biomolecular and Biomedical Research, University College Dublin Belfield, Dublin 4, Ireland; francisca.contreras@ucd.ie (F.C.); werd.al-najim@ucd.ie (W.A.-N.)

**Keywords:** obesity, weight management, nutritional therapy, weight loss, health gain

## Abstract

Introduction: Obesity management strategies such as caloric restriction, very-low-calorie diets (VLCDs), and meal replacements can lead to moderate short-term weight loss. However, many patients face significant challenges in maintaining these results. Personalized interventions, including behavioral counseling and physical activity, have been shown to improve long-term adherence and success. Current clinical guidelines emphasize the pivotal role of dietitians in enhancing patient outcomes through nutritional therapy. When combined with pharmacotherapy and bariatric surgery, the focus shifts from mere weight loss to broader health improvements. Methods: This review explores the evolving role of dietitians in obesity management, advocating for a shift from a weight-centric approach to a more holistic model that prioritizes overall health gains. Key areas of interest include dietetic interventions’ impact on metabolic health, cardiovascular function, gut microbiome balance, inflammation, and psychological well-being. Results: Dietetic interventions have been shown to provide significant health improvements beyond weight loss. These include enhanced metabolic and cardiovascular health, better gut microbiome balance, reduced inflammation, improved sleep quality, mental well-being, and overall quality of life. By focusing on non-scale victories such as improved insulin sensitivity, lipid profiles, and mental health, dietitians play a crucial role in driving long-term success in obesity management. These outcomes highlight the need to shift the focus from short-term weight loss to a more comprehensive view of health gains. Conclusions: The role of dietitians in obesity management is expanding to encompass a more comprehensive and individualized approach. Moving beyond a focus on weight reduction, this paradigm promotes long-term, patient-centered strategies that address the multifactorial nature of obesity. By combining dietary changes with regular physical activity and behavioral support, dietitians can contribute to sustained health improvements, treating obesity as a chronic, complex disease.

## 1. Introduction

Current nutritional therapies and dietetic interventions for weight management employ various approaches designed to promote sustainable weight loss and long-term maintenance. The success of these approaches varies, with dietitians typically focusing on calorie restriction, which can be achieved through different strategies such as macronutrient manipulation, structured diet plans, and meal replacements [1] (Table 1).

Despite their widespread use, these approaches generally show modest weight reduction. Most diets tend to achieve 4–10% weight loss within 6–12 months, with more structured and restrictive diets, like meal replacements and very-low-calorie diets (VLCDs), reaching up to 15% weight loss [2,3,4]. However, long-term maintenance of this weight loss remains challenging and requires ongoing support, physical activity counselling, and behavioural interventions to prevent weight regain [1,3].

**Table 1:** Current dietetic approaches for obesity management.

Diet	Description	Weight Loss	Period	Maintenance
Caloric deficit	Reduction of 500–1000 kcal/day	5–10%	6–12 months	Effective in the short term, but long-term maintenance is challenging without ongoing support [1,3].
Very-low-calorie diets (VLCDs)	800 kcal/day or less	10–15%	12 weeks	Maintaining weight loss is challenging unless VLCDs are followed by structured refeeding and behavioural interventions [3,5]
Low-fat diets	Reducing fat intake to below 30% of total daily energy	3–5%	12 months	Variable success in long-term maintenance, with better outcomes when combined with exercise and lifestyle changes [3,6]
Low-carbohydrate diets	Reducing carbohydrate intake to below 40% of total daily energy	5–7%	12 months	Long-term studies show weight regain in many patients unless the diet is strictly sustained [6]
Low-carbohydrate and high-protein diets	Promoting satiety and reducing insulin levels	5–7%	6–12 months	Long-term adherence is essential for maintenance, and weight regain is common once the diet is no longer followed [2,3]
Mediterranean diet	Rich in fruits, vegetables, whole grains, and healthy fats (primarily olive oil)	4–7%	12 months	Long-term adherence is crucial, and maintenance tends to be more successful due to the diet’s flexibility and emphasis on whole, unprocessed foods [3,5].
Dash diet(Dietary Approaches to Stop Hypertension)	Originally designed to lower blood pressure, adapted for weight loss	3–5%	12 months	Long-term studies show modest weight regain, with successful maintenance requiring ongoing dietitian support [2,3]
Meal replacement programs	Replacing one or two meals per day with especially formulated replacements	8–10%	6 months	Long-term maintenance depends on transitioning to a balanced diet with continued behavioural support [2,3]

Sustained success in weight management depends heavily on continuous counselling and behavioral support. Combining nutritional therapy with behavioural interventions, such as cognitive behavioural therapy (CBT) and motivational interviewing, has been shown to improve adherence and facilitate long-term weight maintenance [7,8]. Studies show that patients who receive ongoing dietitian support are more likely to maintain a 5–10% weight loss over 1–2 years. Regular follow-ups with dietitians after the initial weight loss have also been proven to prevent weight regain, helping individuals maintain a 5–10% loss for up to 2 years [8,9].

All these strategies show a higher success rate when the dietetic approach is tailored to the individual’s specific needs and context. Personalised plans contribute to better adherence, which in turn helps with the maintenance of weight loss. However, weight regain remains a significant challenge in most dietetic interventions, underscoring the need for continued support and behavioural adjustments to ensure long-term success [6,7].

**Figure 1:**
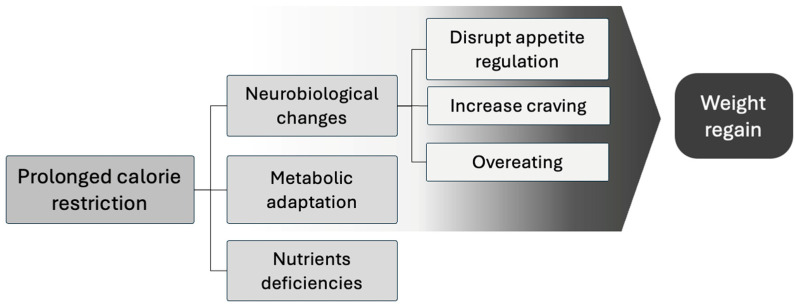
Consequences of prolonged calorie restriction.

While dietitians employ various strategies to help patients manage obesity, evidence highlights significant challenges in maintaining long-term weight loss. These challenges include weight regain after 12 months, difficulty in sustaining adherence to dietary plans, and the ongoing need for personalized interventions and support. Prolonged calorie restriction not only risks causing nutrient deficiencies, which can worsen existing health conditions, but also triggers neurobiological changes that promote weight regain by disrupting appetite regulation and increasing cravings, leading to overeating [7,9,10]. Additionally, metabolic adaptation—a process in which the body’s energy expenditure decreases more than expected after weight loss—further complicates weight maintenance, making it harder to sustain long-term results [11] (Figure 1).

An additional challenge to providing sustained nutritional counselling is financial and systemic barriers. Many patients stop counselling not due to a lack of interest but because of limited insurance coverage or financial constraints. Insufficient coverage for extended sessions can hinder long-term follow-up, increasing the risk of weight regain as patients struggle to maintain dietary and behavioral changes [5,12]. Clarifying patients’ expectations regarding the number of visits and the overall scope of the program before treatment begins can help improve adherence. By setting realistic expectations and planning for the necessary number of follow-up sessions, patients are more likely to commit to the full course of counselling, reducing this issue as a barrier to successful treatment.

## 2. Current Guidelines for Nutritional Therapy in Weight Management

Nutritional therapy plays a pivotal role in weight management. While it may have limited success in achieving significant weight loss when implemented as an independent approach, it becomes an essential component when combined with pharmacotherapy and bariatric surgery [1,3].

### 2.1. Nutritional Therapy

The current guidelines for nutritional therapy in weight management consider the following four key elements:

*Personalised intervention:* The European Association for the Study of Obesity (EASO) emphasizes Medical Nutrition Therapy (MNT), which involves creating individualised dietary interventions that balance caloric reduction with high dietary quality, and suggests that macronutrient distribution should be flexible to match personal preferences for long-term adherence [1,9].

*Energy Deficit:* Guidelines from the EASO, the Association for the Study of Obesity on the Island of Ireland (ASOI), and the Brazilian Association for the Study of Obesity and Metabolic Syndrome (ABESO) all recommend a daily caloric reduction of 500–1000 kcal. This targeted energy deficit aims to achieve a sustainable weight loss of approximately 0.5–1 kg per week, offering a manageable approach to long-term weight management [1,10,13].

*Balanced Macronutrient:* While various macronutrient ratios can be effective, patient adherence is critical. A diet that can be sustained over time is more important than a specific macronutrient breakdown [1,14]. The Preventing Overweight Using Novel Dietary Strategies (POUNDS Lost) Study has shown that diverse dietary patterns can achieve similar weight loss outcomes as long as they promote caloric restriction [14].

*Food quality:* Guidelines from the EASO and the ABESO emphasize the importance of choosing nutrient-dense foods like fruits, vegetables, whole grains, lean proteins, and healthy fats. Minimally processed foods should be prioritised to maintain satiety and improve overall health [1,13].

### 2.2. Nutritional Intervention in Pharmacotherapy

Pharmacotherapy, using medications like glucagon-like peptide-1 (GLP-1) agonists, helps to reduce appetite and increase satiety, making it easier for patients to adhere to reduced-calorie diets [3]. Another type of medication, known as lipase inhibitors, works by blocking gastric and pancreatic lipase, which reduces the amount of fat absorbed from the diet [2]. Nutritional therapy serves as a foundational component of pharmacotherapy, enhancing patients’ health outcomes beyond weight loss. It supports patients through the following: (a) guiding them toward healthier eating patterns [2,3]; (b) managing potential gastrointestinal side effects [2,3] (Table 2); and (c) preventing nutritional deficiencies [2,3]. This comprehensive approach ensures that patients maximize the benefits of pharmacological interventions while promoting overall well-being.

While pharmacotherapy is effective, it does not eliminate the need for dietary changes. Although the medication reduces the pressure on patients to actively lower their calorie intake by naturally curbing appetite, the role of the dietitian becomes even more critical in ensuring the quality of food choices. Dietitians must ensure patients receive adequate nutrition despite a reduced appetite and lower calorie intake. Additionally, they should work closely with the medical team to determine the optimal medication dose, which may not necessarily be the highest, but rather the one where the patient feels their best, consuming a moderate amount of healthy calories while still achieving weight loss or maintenance. Therefore, nutritional guidance remains essential to maximizing the long-term effectiveness of pharmacotherapy in weight management [3].

### 2.3. Nutritional Intervention in Bariatric Surgery

Bariatric surgery is the most effective weight loss strategy. However, nutritional intervention is crucial both pre- and post-surgery to ensure successful outcomes and prevent surgical complications [1,16]. The goals of nutritional therapy in bariatric surgery include the following: (a) guiding the patient to follow a very low-calorie diet to reduce liver size pre-surgery and optimize surgical outcomes [1,16]; (b) helping the patient develop healthier, sustainable eating habits [1]; (c) supporting post-operative healing through a progressive diet [1,16]; and (d) preventing malnutrition and ensuring proper supplementation of essential nutrients [1,16].

### 2.4. Limitation of These Approaches

**Figure 2:**
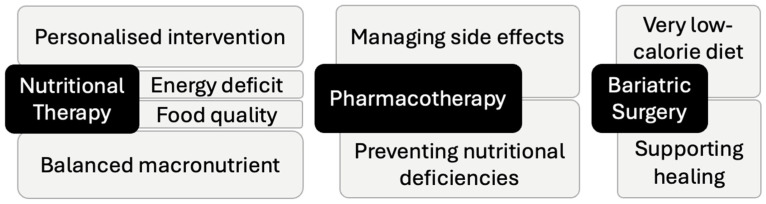
Role of nutritional therapy in weight management. The figure illustrates the central role of nutritional therapy, whether used alone or in combination with pharmacotherapy or bariatric surgery, where its role adapts to complement each treatment. While these approaches target different aspects of weight loss, nutritional therapy remains fundamental for sustained effectiveness, improved outcomes, and overall health across all treatment methods.

The role of dietitians extends far beyond simple weight loss, significantly influencing lifestyle changes, improving the quality of life, and preventing the complications that arise from weight management therapies (Figure 2). However, there remains a notable lack of standardized guidelines on how to approach diverse weight loss strategies and effectively measure improvements in non-weight-related outcomes. Establishing robust frameworks to evaluate the efficacy of these interventions could improve treatment outcomes.

## 3. New Approach: Health Gain Beyond Weight Loss

**Figure 3:**
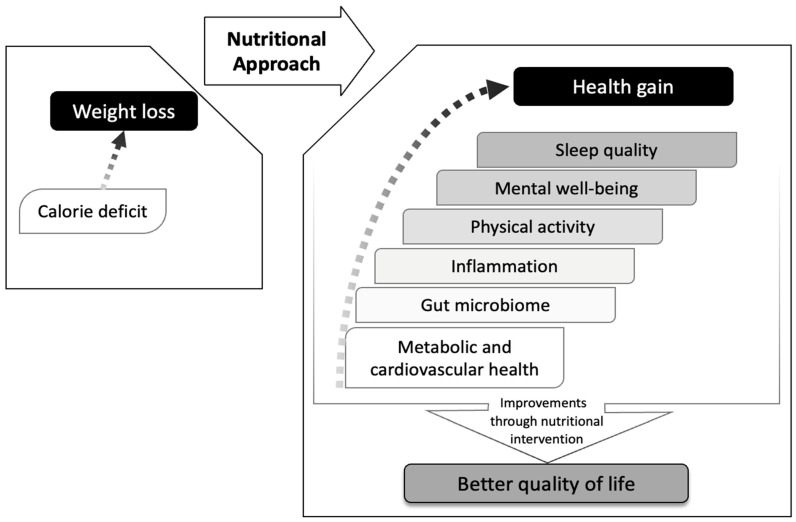
Shifting dietary approach from weight loss to health gain. This figure illustrates the transition from a traditional focus on calorie deficit-driven weight loss to a more comprehensive approach targeting overall health improvements. On the left, weight loss is presented as the primary outcome of a calorie deficit. On the right, the figure expands to highlight multiple health gains achieved through nutritional interventions, such as improved sleep quality, mental well-being, increased physical activity, reduced inflammation, a healthier gut microbiome, and enhanced metabolic and cardiovascular health. These health gains collectively contribute to a better quality of life. This paradigm shift encourages the adoption of dietary strategies that prioritise long-term health and well-being over short-term weight loss.

In this review, we advocate for redefining the dietitian’s role by shifting the focus from primarily achieving a calorie deficit for weight loss to adopting a more holistic approach that prioritizes overall health improvements (Figure 3). While calorie reduction remains a key component of weight management, success in treatment should not be defined by weight loss alone. Instead, standardized evaluation metrics should include other important health outcomes, such as metabolic health, cardiovascular function, mental well-being, and quality of life. Recent research increasingly supports this approach, demonstrating that dietary interventions can yield significant health benefits beyond weight loss [2,13,14].

After all, obesity is ‘a chronic complex disease defined by excessive fat deposits that can impair health’ [17]; therefore, the treatment should also focus on mitigating the associated health impairments rather than focusing solely in the excessive fat adiposity reduction [5,9,11,18,19,20].

### 3.1. Metabolic and Cardiovascular Health

Metabolic parameters are one of the most improved elements through dietetic intervention. Depending on the baseline condition, low-carb or low-fat diets or patterns such as Mediterranean or Dietary Approaches to Stop Hypertension (DASH) diets can have a profound impact on various metabolic and cardiovascular health indicators [2,3,5,6,13,19,20].

The Mediterranean diet, rich in monounsaturated fats from olive oil, nuts, and fish, has been shown to reduce LDL cholesterol and improve HDL cholesterol levels, leading to a more favorable lipid profile and reduced cardiovascular risk [5,19]. The DASH diet, which emphasizes fruits, vegetables, and low-fat dairy products, is particularly effective in reducing blood pressure, likely due to its high potassium and magnesium content, along with its low sodium intake [6,13]. These improvements in blood pressure contribute directly to better endothelial function, a key marker of cardiovascular health [13]. Furthermore, both the DASH and Mediterranean diets are associated with reduced arterial stiffness, which decreases the risk of atherosclerosis and other cardiovascular events [2,6,19].

In addition to these cardiovascular benefits, dietary interventions also impact metabolic hormones. Contrary to the effect of a prolonged caloric restriction that can lead to disrupted appetite regulation, increased cravings and, ultimately, increased food intake (Figure 1) [7,9,10,11], certain dietetic approaches can aid in improving leptin signalling [1]. Diets with a low glycemic index or those high in protein can help regulate appetite by improving leptin sensitivity and promoting fat loss from visceral adipose tissue, which is often associated with insulin resistance [1], helping to maintain stable insulin levels thus reducing cravings for high-calorie foods and improving overall metabolic health [3,9,18]. On the other hand, diets high in protein and fibre can also attenuate post-meal ghrelin spikes, reducing ghrelin levels and contributing to prolonged feelings of satiety and contributing to better weight management and metabolic health [7].

By targeting the hormonal dysregulation common in obesity and the cardiovascular risks associated with metabolic syndrome, these dietary strategies can promote sustainable health improvements, lower the risk of metabolic diseases, and improve quality of life.

### 3.2. Gut Microbiome

The trillions of microorganisms that compose the gut microbiome plays a crucial role in regulating digestion, immune function, and energy metabolism. Specific dietary patterns can shift the composition and function of the gut microbiota, leading to improvements in metabolic processes that are independent of weight loss [21,22]. Dietary interventions that increase fibre intake enhance short-chain fatty acid (SCFA) production, such as acetate, propionate, and butyrate. SCFAs serve as an energy source for colon cells that improves metabolic pathways [21,22,23,24,25].

SCFAs’ influence in gluconeogenesis, promotes improved glucose tolerance and reduced fat accumulation, thereby increasing bile acid metabolism, which is critical for fat digestion, and positively impacting lipid metabolism [22,23]. These metabolites regulate fat storage and energy expenditure by favouring the growth of beneficial bacteria [23]. Additionally, SCFAs modulate the production of hormones involved in appetite regulation, such as peptide YY (PYY) and GLP-1, which contribute to enhanced satiety and energy balance [25].

### 3.3. Inflammation

Chronic inflammation, defined as a prolonged, low-grade inflammatory response that persists over time, is characterized by elevated levels of circulating pro-inflammatory markers such as C-reactive protein (CRP), interleukin-6 (IL-6), and tumor necrosis factor-alpha (TNF-α). Clinically, these biomarkers are often measured to assess the degree of systemic inflammation in humans, particularly in conditions like type 2 diabetes, cardiovascular disease, and metabolic syndrome. Chronic inflammation contributes to the progression of these diseases by promoting insulin resistance, endothelial dysfunction, and plaque formation in the arteries [2,3].

Dietary interventions that focus on nutrient quality and gut microbiome modulation have been shown to reduce this chronic inflammation [2,3].

High-fibre diets, which increase SCFA production are particularly effective in reducing inflammation through inhibiting the production of pro-inflammatory cytokines, such as tumour necrosis factor-alpha (TNF-α), and promoting the development of regulatory T cells, which are essential for maintaining immune homeostasis [21,22]. Furthermore, SCFAs improve gut barrier function and reduce intestinal permeability, thereby preventing endotoxin translocation which triggers systemic inflammation [24].

In addition to fibre, diets rich in omega-3 fatty acids, polyphenols, and other bioactive compounds also play a crucial role in reducing inflammation. Omega-3 fatty acids have been shown to decrease the production of inflammatory eicosanoids and cytokines [2,4]. Polyphenols, found in fruits, vegetables, and whole grains, exert anti-inflammatory effects by modulating the nuclear factor-kappa B (NF-κB) pathway, which is a critical regulator of inflammatory responses [4,23].

### 3.4. Sleep Quality

Obesity-related factors such as chronic inflammation, insulin resistance, and increased appetite-regulating hormones, such as ghrelin, contribute to disrupted sleep patterns, including obstructive sleep apnoea (OSA) [7,9]. These disturbances are exacerbated by poor sleep quality, which itself can promote weight gain through dysregulated appetite control, leading to increased cravings for high-calorie, nutrient-poor foods [7,26,27]. This cycle perpetuates obesity by fostering unhealthy eating behaviours, increasing fatigue, reducing physical activity, and encouraging a sedentary lifestyle [7,9].

Nutritional interventions that promote balanced macronutrient intake, particularly by increasing fibre and lowering glycaemic index, can enhance sleep quality independently of weight reduction [28,29,30]. Micronutrients like magnesium, zinc, and melatonin supplements have shown to enhance sleep quality by regulating the sleep–wake cycle and reducing sleep onset latency [1,22]. Diets that are high in fibre and low in sugar and saturated fats are associated with deeper and more restorative sleep, whereas high-sugar diets result in lighter, less restorative sleep [29,30]. The Mediterranean diet has been associated with improved sleep quality due to its high content of antioxidants, omega-3 fatty acids, and anti-inflammatory properties [27,31].

### 3.5. Mental Well-Being

Dietary interventions have been shown to positively impact on mental well-being by influencing the gut–brain axis, promoting favourable changes in mood, cognitive function, and stress resilience. The SCFAs produced by the gut microbiota play a critical role in this process by interacting with the nervous system and modulating the production of mood-regulating hormones such as serotonin [32]. Mediterranean diets have been associated with reduced symptoms of depression and anxiety, due to their anti-inflammatory properties, reduction in oxidative stress, and promotion of a diverse gut microbiota [31]. Omega-3 fatty acids, found in fish and nuts, are also known to support brain function and reduce symptoms of mood disorders by modulating neuroinflammation [33]. Moreover, specific nutrients such as vitamins B, D, magnesium, and polyphenols from plant-based foods have been linked to improved stress response, enhanced serotonin production, and reduced cortisol levels [34].

### 3.6. Physical Activity

Physical activity provides significant benefits for weight management, offering numerous health benefits beyond weight loss [20,35]. Engaging in physical activity improves metabolic function and reduces cardiovascular risk by increasing insulin sensitivity, enhancing glucose uptake, lowering blood pressure, and improving lipid profiles, even without significant weight loss [36,37,38]. During weight loss, physical activity, particularly resistance training, helps preserve lean muscle mass and improve bone density while primarily reducing fat mass [1,5,37]. Regular physical activity enhances mental health by reducing symptoms of depression and anxiety, improving mood, and boosting overall well-being [31,33]. It also helps lower stress levels, which is particularly important for weight management since stress can trigger overeating and poor dietary habits [34].

While patients might believe the primary goal of physical activity is to create a larger calorie deficit, evidence suggests that people often compensate for the calories burned by eating more post-exercise [39]. Therefore, discussions with patients should shift away from exercise as a tool for burning calories and instead emphasize the wide-ranging health benefits, such as improved metabolic health, mental well-being, and enhanced quality of life [35].

Physical activity also plays a role in regulating appetite by modulating hunger-related hormones such as ghrelin and leptin [7]. Moreover, physical activity improves quality of life by enhancing physical functioning, mobility, and energy levels while reducing the risk of injury [36]. These improvements make daily activities easier to manage and support long-term adherence to healthier lifestyle habits [20].

Given these comprehensive benefits, it is essential for dietitians to celebrate small achievements with patients, such as beginning a regular walking routine or incorporating 10–15 min of exercise into their day. These wins can boost confidence and motivation. However, dietitians should also challenge patients when appropriate, balancing encouragement with a realistic understanding of each individual’s limitations. Additionally, dietary interventions should complement physical activity by supporting muscle preservation and bone health through the adequate intake of protein, calcium, and vitamin D [1,13].

### 3.7. Quality of Life

Improvement in quality of life (QoL) is a critical measure when evaluating the success of dietary interventions, often assessed through health-related quality of life (HRQoL) questionnaires. These tools efficiently gather data on individuals’ physical functioning, psycho-social well-being, and emotional health. Although research specifically focused on the relationship between dietary interventions and QoL is somewhat limited, existing evidence suggests that well-balanced diets, particularly the Mediterranean diet, offer significant benefits [31,34,40,41,42,43].

The Mediterranean diet is associated with better physical functioning, vitality, emotional well-being, and social functioning [31,41,43]. These benefits extend beyond weight management, contributing to improved cognitive function, reduced risk of depression, and enhanced emotional resilience [33]. Additionally, dietary interventions that prioritize gut health have been shown to enhance quality of life (QoL) by fostering a healthy gut microbiome. This contributes to improved immune function, mental well-being, and increased energy levels [21,25,32].

Despite the limited association between dietary intervention and improvement in quality of life, it can be assumed that any well-balanced diet tailored to individual needs, which raises energy levels, reduces chronic disease symptoms, and improves emotional health would enhance the quality of life. For example, dietary strategies used in managing type 2 diabetes not only improve glycaemic control but also alleviate fatigue, enhance physical mobility, and improve mood and mental clarity [6,14], all of which are important contributors to QoL.

## 4. Evaluation of Non-Scale Victories

**Figure 4:**
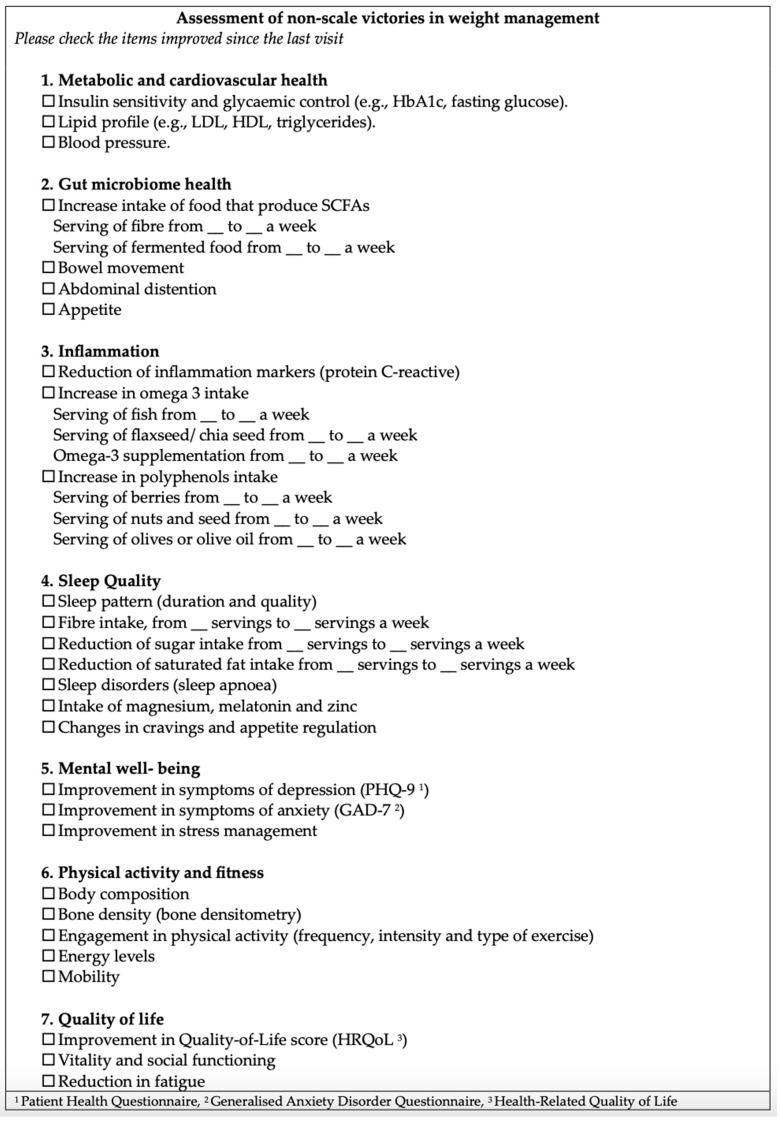
Checklist of non-scale victories in weight management.

We propose that dietitians can assess the effectiveness of dietary interventions in patients with obesity by utilizing a standardized checklist that tracks non-scale victories (NSVs) (Figure 4). This checklist includes key indicators such as improvements in energy levels, sleep quality, mobility, and psychological well-being, offering a comprehensive view of the patient’s progress. By providing a clear visual representation of these improvements, the checklist reinforces a sense of achievement, which can be highly motivating for patients, especially when weight loss is slower or less significant than expected. Focusing on these broader health gains helps patients shift their attention away from the scale and encourages sustained commitment to long-term lifestyle changes.

This checklist was developed to be used by any healthcare professional involved in patient care, with the specific team member conducting the assessment being determined through internal multidisciplinary team discussions.

## 5. Conclusions

In conclusion, current nutritional therapies for weight management emphasize a variety of approaches, ranging from calorie restriction to structured diets like VLCDs and meal replacement programs. While these interventions show initial success in achieving weight loss, maintaining that progress remains challenging. Evidence underscores the importance of personalized and long-term support through behavioural counselling and physical activity to sustain results. However, shifting the focus from weight loss alone to overall health gains—such as improvements in metabolic health, gut microbiome, inflammation, sleep quality, mental well-being, and quality of life—presents a more holistic and sustainable path forward. Integrating non-scale victories as markers of success encourages long-term adherence, helping patients make lasting lifestyle changes. Ultimately, this comprehensive approach addresses the complex nature of obesity and enhances both physical and mental health outcomes.

## Figures and Tables

**Table 2:** Nutritional strategies to manage common gastrointestinal side effects of pharmacotherapy in weight loss treatment.

Side Effects	Strategies	Recommendations
Constipation	Increase fibre intake [8,12,15] Increase water intake [12,15] Physical activity [8] Portion control [8,12]	Include high in fibre fruit and vegetables [8,12], such as kiwi, plums, pear, berries, and any leafy green, drink glass of prune juice, add flax or chia seed Stay active throughout the day and after meals [8] Eat small portions add snacks if needed [8,12] Eat slowly [12,15]
Diarrhoea	Increase water intake [8,12,15] Prefer soluble fibre [8] Avoid fatty food [8,15] Portion control [8,12]	Prefer fruit and vegetables [12,15] such as bananas and carrots [8] Avoid butter, cream, sauces and deep-fried food [8] Reduced portion intake, add snacks if needed [8,12]
Reflux/Nausea	Increase water intake [8,12,15] Eat slowly [8] Avoid lying down after meals [8,12] Avoid fatty food, carbonated drinks and caffeine [8,12,15] Portion control [8,12,15]	Eat just when you are hungry [8,15] Avoid eating too late in the evening [8,12] Prefer a lighter meal in the evening time [12] Drink plenty of water [8,15] Drink ginger and mint tea [8] Avoid thick creamy textured food, fizzy drinks and caffeine [8,12] Reduced portion intake, add snacks if needed [8,12,15]
